# Supplementary material for: Validation and Psychometric Properties of the Spanish Version of the Fear of Childbirth Questionnaire (CFQ-e)
Source: J Clin Med. 2022 Mar 26;11(7):1843. doi: 10.3390/jcm11071843 (PMC8999905; doi:10.3390/jcm11071843)
Supplement: Supplementary file 1 [file jcm-11-01843-s001.zip › TABLE S1. First Version of CFQ-e (spanish version).pdf]

Cuestionario de miedo al parto-CFQ-e VERSIÓN PRIMERA. A cumplimentar por la gestante

Gracias por colaborar en este estudio. A continuación se adjunta una serie de situaciones del parto y nacimiento que a algunas mujeres les pueden generar miedo o terror. Para cada una delas situaciones reseñadas, indique cuanto miedo le genera (marque con un cruz o círculo). Use esta escala como guía:

Nada en absoluto	0 No tengo miedo en absoluto de este aspecto del parto.
Un poco	1 Tengo algo de miedo a este aspecto del parto.
Moderadamente	2 Tengo bastante miedo a este aspecto del parto.
Mucho	3 Tengo mucho miedo a este aspecto del parto.
Extremadamente	4 Tengo un miedo extremo a este aspecto del parto.

**SITUACIÓN**	**MIEDO**				
	Nada en absoluto	Un poco	Moderadamente	Mucho	Extremo
Cuanto miedo te genera…					
1. Que te hagan daño por una asistencia médica incompetente ?	0	1	2	3	4
2. Sufrir un desgarro o daño rectal como consecuencia del parto?	0	1	2	3	4
3. Morir durante el parto?	0	1	2	3	4
4. Recibir anestesia general?	0	1	2	3	4
5. Que te administren inyecciones?	0	1	2	3	4
6. Que dañen o perjudiquen al bebé como consecuencia del parto?	0	1	2	3	4
7. Que otras personas te vean desnuda durante el parto?	0	1	2	3	4
8. Sufrir un desgarro vaginal durante el parto?	0	1	2	3	4
9. Que el bebé sufra algún daño durante el parto?	0	1	2	3	4
10. Que dañen al bebe en una intervención médica durante el parto (ej. ventosa, anestesia, forceps...)?	0	1	2	3	4

11. Que no te hagan una cesárea, cuando es lo que tu prefieres?	0	1	2	3	4
12. Que tu vagina se estire por tener un parto vaginal?	0	1	2	3	4
13. Disfrutar menos de las relaciones sexuales por el estiramiento de la vagina a causa del parto vaginal?	0	1	2	3	4

**SITUACIÓN**	**MIEDO**				
Cuanto miedo te genera ...	Nada en absoluto	Un poco	Moderadamente	Mucho	Extremo Mucho
14. Perder el control de tus emociones frente a otras personas (ser maleducada, gritar) durante el parto?	0	1	2	3	4
15. Que tu cuerpo sea menos atractivo tras el parto?	0	1	2	3	4
16. Que el bebé se asfixie durante el parto?	0	1	2	3	4
17. Necesitar un forceps o ventosa?	0	1	2	3	4
18. No recibir la medicación para el dolor que necesites?	0	1	2	3	4
19. Que te realicen una episiotomía (que te realicen un corte en tu vagina)?	0	1	2	3	4
20. Que el bebé se muera durante el parto?	0	1	2	3	4
21. Que otras personas te vean orinar durante el parto?	0	1	2	3	4
22. Que te administren la epidural?	0	1	2	3	4
23. Sentirte observada por desconocidos durante el parto?	0	1	2	3	4
24. Que tu vagina se vea menos atractiva tras un parto vaginal?	0	1	2	3	4
25. Que te queden cicatrices tras la cesárea?	0	1	2	3	4
26. Disfrutar menos de las relaciones sexuales por sentir dolor o molestias tras	0	1	2	3	4
27. Que tu pareja disfrute menos de las relaciones sexuales después del parto por el estiramiento de tu vagina tras el parto	0	1	2	3	4
28. Necesitar puntos de sutura tras el parto?	0	1	2	3	4
29. Que no te pongan la epidural durante el parto en el caso de quererla o necesitarla?	0	1	2	3	4
30. Sentir dolor durante el parto?	0	1	2	3	4

31. Tener un parto vaginal?	0	1	2	3	4
32. Que otras personas te vean defecar durante el parto ?	0	1	2	3	4
33. No poder tener un parto via vaginal a pesar de ser lo que prefieres?	0	1	2	3	4

SITUACIÓN	MIEDO				
	Nada en absoluto	Un poco	Moderadamente	Mucho	Extremo
Cuanto miedo te genera ...					
34. Sentir dolor mientras empujas al bebé?	0	1	2	3	4
35. Sentir dolor durante un parto vía vaginal?	0	1	2	3	4
36. Tener una cesárea?	0	1	2	3	4
37. Sentir dolor durante las contracciones?	0	1	2	3	4
38. Que te sonden (un tubo que se inserta en la uretra para recoger la orina)?	0	1	2	3	4
39. Sentir dolor durante una cesárea?	0	1	2	3	4
40. No poder tener el tipo de parto que te gustaría (por ejemplo: vaginal o cesárea)?	0	1	2	3	4
Otros (especifica):___________________	0	1	2	3	4

ESCALA DE INTERFERENCIA

Algunas veces nuestros miedos pueden afectar en nuestras vidas; por ejemplo en nuestro trabajo, familia u otras cosas que nos gusta hacer. Indique, utilizando la siguiente escala, cuánto afectansus miedos al parto en tu vida:

Ninguna interferencia	0	Mi miedo no interfiere en ningún aspecto de mi vida.
Interferencia leve	1	Mi miedo interfiere en mi vida, pero solo en pequeñas cosas.
Interferencia moderada	2	Mi miedo interfiere en algunas actividades que son importantes para mí. Definitivamente mi miedo causa problemas en mi vida.
Interferencia severa	3	Mi miedo me impide que haga cosas importantes en mi vida. (ejemplo, acudir a citas médicas importantes, trabajo, cuidado de mis hijos).
Interferencia extrema	4	Mi miedo me impide que haga muchas actividades importantes. Mi miedo causa serios problemas en mi vida.

ESFERA DE LA VIDA	INTERFERENCIA				
	Ninguna	Leve	Moderad	Severa	Extrema
1. En la relación con mi pareja o marido.	0	1	2	3	4
2. En la relación con mi familia.	0	1	2	3	4
3. En otras relaciones de mi vida	0	1	2	3	4
4. En la relación con los profesionales sanitarios encargados del control de mi embarazo	0	1	2	3	4
5. En mi vida laboral	0	1	2	3	4
6. En mis actividades de ocio	0	1	2	3	4
7. En la preparación para la llegada de mi bebé	0	1	2	3	4
8. Otros (especifica): ___________________	0	1	2	3	4

INSTRUCCIONES DE PUNTUACIÓN CFQ-E PRIMERA VERSIÓN

CFQ-e PUNTUACIÓN TOTAL: Sumar la puntuación de los 40 ítems

Puntuación en las Subescalas: Para cada una de las nueve subescalas enumeradas a continuación, sume los elementos de la subescala. Para crear la puntuación media (para poder comparar entre subescalas), divida la puntuación de la subescala por el número de elementos de la misma.

SUBESCALA MIEDO A LA PÉRDIDA DE PLACER/ATRACTIVO SEXUAL

12-Que tu vagina se estire por tener un parto vaginal
13-Disfrutar menos de las relaciones sexuales por el estiramiento de la vagina a causa del parto vaginal
15-Que tu cuerpo sea menos atractivo tras el parto
24-Que tu vagina se vea menos atractiva tras un parto vaginal
26-Disfrutar menos de las relaciones sexuales por sentir dolor o molestias tras el parto
27-Que tu pareja disfrute menos de las relaciones sexuales después del parto por el estiramiento de tu vagina tras el parto

SUBESCALA MIEDO AL DOLOR DE UN PARTO VAGINAL

30-Sentir dolor durante el parto
31-Tener un parto vaginal
34-Sentir dolor mientras empujas al bebe
35-Sentir dolor durante un parto vía vaginal
37-Sentir dolor durante las contracciones

SUBESCALA MIEDO A LAS INTERVENCIONES MÉDICAS

1-Que te hagan daño por una asistencia médica incompetente
4-Recibir anestesia general
5-Que te administren inyecciones
22-Que te administren la epidural
25-Que te queden cicatrices tras la cesárea
38-Que te sonden (un tubo que se inserta en la uretra para recoger la orina
39-Sentir dolor durante una cesárea

SUBESCALA MIEDO A SENTIRSE AVERGONZADA

7 Que otras personas te vean desnuda durante el parto
14-Perder el control de tus emociones frente a otras personas (ser maleducada, gritar) durante el parto
21-Que otras personas te vean orinar durante el parto
23-Sentirte observada por desconocidos durante el parto
32-Que otras personas te vean defecar durante el parto

SUBESCALA MIEDO A DAÑOS AL BEBÉ

6-Que dañen o perjudiquen al bebe como consecuencia del parto
9-Que el bebe sufra algún daño durante el parto
10-Que dañen al bebe en una intervención médica durante el parto (ej, ventosa, anestesia, fórceps ...)

SUBESCALA MIEDO A LA CESÁREA

33-No poder tener un parto vaginal a pesar de ser lo que prefieres
36-Tener una cesárea
40-No poder tener el tipo de parto que te gustaría (por ejemplo vaginal o cesárea)

SUBESCALA MIEDO A QUE LA MADRE O EL BEBE SE MUERAN

3-Morir durante el parto
16-Que el bebe se asfixie durante el parto
20-Que el bebe muera durante el parto

SUBESCALA MIEDO A RECIBIR MEDICACIÓN INSUFICIENTE PARA EL DOLOR

11-Que no te hagan una cesárea cuando es lo que tú quieres
18-No recibir la medicación para el dolor que necesitas
29-Que no te pongan la epidural durante el parto en el caso de quererla o necesitarla

SUBESCALA MIEDO AL DAÑO CORPORAL POR UN PARTO VAGINAL

2-Sufrir un desgarro o daño rectal como consecuencia del parto
8-Sufrir un desgarro vaginal durante el parto
17-Necesitar un fórceps o ventosa
19-Que te realicen una episiotomía (que te realicen un corte en tu vagina)
28-Necesitar puntos de sutura tras el parto
