# Supplementary material for: Validation and Psychometric Properties of the Spanish Version of the Fear of Childbirth Questionnaire (CFQ-e)
Source: J Clin Med. 2022 Mar 26;11(7):1843. doi: 10.3390/jcm11071843 (PMC8999905; doi:10.3390/jcm11071843)
Supplement: Supplementary file 1 [file jcm-11-01843-s001.zip › TABLE S10. CFQ-e Reliability scores.pdf]

**Fear of Childbirth Questionnaire (CFQ)**<br>Original model<br>40 items<br>9 subscales			**Fear of Childbirth Questionnaire Spanish version (CFQ-e)**<br>Spanish version after validation<br>37 items<br>4 subscales		
	Cronbach's Coefficient	Omega Index		Cronbach's Coefficient	Omega Index
Total	**0.951**	**0.949**	Total	**0.947**	**0.945**
Fear of loss of sexual pleasure /attractiveness (6 items)	0.888	0.890	Fears relating to sexual aspects and embarrassment (10 items)	0.888	0.894
Fear of Pain from a vaginal birth (5 items)	0.900	0.906			
Fear of Medical interventions (7 items)	0.789	0.788	Fear of Medical Interventions (10 items)	0.878	0.878
Fear of Embarrassment (5 items)	0.779	0.796			
Fear of harm to baby (3 items)	0.928	0.929			
Fear of Caesarean Birth (3 items)	0.833	0.833	Fear of pain (7 items)	0.897	0.896
Fear of mom or baby dying (3 items)	0.799	0.804	Fear of harm and dying (10 items)	0.913	0.911
Fear of insufficient pain medication (3 items)	0.737	0.751			
Fear of body damage from a vaginal birth (5 items)	0.831	0.830			

Supplementary Material S10. CFQ-e Reliability scores
