# Supplementary material for: Validation and Psychometric Properties of the Spanish Version of the Fear of Childbirth Questionnaire (CFQ-e)
Source: J Clin Med. 2022 Mar 26;11(7):1843. doi: 10.3390/jcm11071843 (PMC8999905; doi:10.3390/jcm11071843)
Supplement: Supplementary file 1 [file jcm-11-01843-s001.zip › TABLE S2. Profile and CVI-E experts.pdf]

	Profession and Gender	Content Validity Index-Expert (CVI-E)[a]	Profile of the expert
Expert 1	Midwife/Female	0.82	Full-time teacher attached to the Multi-professional Teaching Unit of Obstetrics and Gynaecology. Completed her professional studies in England and contributed to the initial translation of the questionnaire.
Expert 2	Midwife/Female	0.77	PhD. Associate university professor. Conducts her professional activity at the Department of Healthcare Programmes of the Canary Islands Health Service-Women's Services.
Expert 3	Obstetrician/Female	0.75	Responsible for the Obstetric-Gynaecological-Delivery Unit of the XXXX. Chair of the XXXXX Breastfeeding Committee.
Expert 4	Midwife/Male	0.55	Midwife with over 20 years of experience, mostly in the United Kingdom. Selected for his knowledge of the population in which the original questionnaire was validated.
Expert 5	Midwife/Male	0.90	PhD. Associate university professor, working directly in the delivery unit. Doctoral Thesis on childbirth satisfaction and expectations.
Expert 6	Nurse/Male	1	PhD and University Professor. An expert in Research Methodology. Has participated in multiple projects relating to the design, adaptation and validation of Health Science questionnaires.
Expert 7	Nurse/Female	0.52	PhD and full-time associate university professor. Evaluator for the Spanish Ministry of Health's FIS (strategic action in Health). Director of a Scientific Journal indexed in the Scimago Journal Rank (SJR).
Expert 8	Obstetrician/Female	0.72	PhD and Professor linked to the University. Head of the Obstetrics and Gynaecology Unit of XXXXX.
Expert 9	Midwife/Male	0.72	Healthcare Midwife. PhD candidate with a thesis on Fear of Childbirth.
Expert 10	Nurse/Female	1	PhD and University Professor. Experienced in research in the field of Psychology. Editor of a Scientific Journal indexed in the Scimago Journal Rank (SJR).

a= Calculated as Number of items that scored 3-4 points with each expert/Number of total items in the questionnaire (40 items).

Supplementary Table S2. Profile of the experts who contributed to the content validation and each expert's CVI
