# Supplementary material for: Validation and Psychometric Properties of the Spanish Version of the Fear of Childbirth Questionnaire (CFQ-e)
Source: J Clin Med. 2022 Mar 26;11(7):1843. doi: 10.3390/jcm11071843 (PMC8999905; doi:10.3390/jcm11071843)
Supplement: Supplementary file 1 [file jcm-11-01843-s001.zip › TABLE S3. I-CVI 40 items CFQ-e.pdf]

Nº Item	Number of experts who score 3 or 4 points	I-CVI[a]	Pc[b]	K*[c]	Evaluation[d]
**1**	10	1,00	0,001	1,00	Excelent
**2**	9	0,90	0,010	0,90	Excelent
**3**	9	0,90	0,010	0,90	Excelent
**4**	7	0,70	0,117	0,66	Good
**5**	4	0,40	0,205	0,25	Poor
**6**	10	1,00	0,001	1,00	Excelent
**7**	7	0,70	0,117	0,66	Good
**8**	9	0,90	0,010	0,90	Excelent
**9**	10	1,00	0,001	1,00	Excelent
**10**	10	1,00	0,001	1,00	Excelent
**11**	7	0,70	0,117	0,66	Good
**12**	2	0,20	0,044	0,16	Poor
**13**	4	0,40	0,205	0,25	Poor
**14**	6	0,60	0,205	0,50	Fair
**15**	7	0,70	0,117	0,66	Good
**16**	10	1,00	0,001	1,00	Excelent
**17**	9	0,90	0,010	0,90	Excelent
**18**	10	1,00	0,001	1,00	Excelent
**19**	9	0,90	0,010	0,90	Excelent
**20**	10	1,00	0,001	1,00	Excelent
**21**	6	0,60	0,205	0,50	Fair
**22**	4	0,40	0,205	0,25	Poor
**23**	7	0,70	0,117	0,66	Good
**24**	6	0,60	0,205	0,50	Fair
**25**	7	0,70	0,117	0,66	Good
**26**	9	0,90	0,010	0,90	Excelent
**27**	7	0,70	0,117	0,66	Good
**28**	7	0,70	0,117	0,66	Good
**29**	10	1,00	0,001	1,00	Excelent
**30**	9	0,90	0,010	0,90	Excelent

**31**	7	0,70	0,117	0,66	Good
**32**	6	0,60	0,205	0,50	Fair
**33**	10	1,00	0,001	1,00	Excelent
**34**	9	0,90	0,010	0,90	Excelent
**35**	9	0,90	0,010	0,90	Excelent
**36**	10	1,00	0,001	1,00	Excelent
**37**	7	0,70	0,117	0,66	Good
**38**	4	0,40	0,205	0,25	Poor
**39**	8	0,80	0,044	0,79	Excelent
**40**	10	1,00	0,001	1,00	Excelent

[a] I-CVI = Item-level content validity index
[b] Pc: Probability of chance agreement Pc= [N!/(A!(N-A)!)] *0.5^N. where N = nº expert and A = n of agreement with good relevance
[c] K*: Modified kappa coefficient designating K* = (I-CVI- Pc)/ (1- Pc).
[d] Evaluation criteria for kappa. K*: Poor K*< 0,40; Fair K*= 0,40-0,599 ; Good K*= 0,60-0,749 ; Excelent: K* >0,75.

Supplementary Table S3. I-CVI scores for each ítem of CFQ-e.
