# Supplementary material for: Validation and Psychometric Properties of the Spanish Version of the Fear of Childbirth Questionnaire (CFQ-e)
Source: J Clin Med. 2022 Mar 26;11(7):1843. doi: 10.3390/jcm11071843 (PMC8999905; doi:10.3390/jcm11071843)
Supplement: Supplementary file 1 [file jcm-11-01843-s001.zip › TABLE S4. Interference Scale.pdf]

How fear affects/Sphere of life	Floor No interference[a] n(%)	Ceiling Extreme interference[a] n(%)	M(SD)
1.With my relationship with my partner/spouse.	327 (58.7%)	7 (1.3%)	0.64 (0.90)
2.With my relationship with my family members	346 (62.1%)	5 (0.9%)	0.60 (0.90)
3.With other relationships in my life	330 (59. 2%)	2 (0.4%)	0.59 (0.82)
4.With my relationships with my prenatal caregivers	289 (51.9%)	6 (1.1%)	0.72 (0.89)
5.With my work life	277 (49.7%)	9 (1.6%)	0.77 (0.95)
6.With my leisure activities	302 (54.2%)	3 (0.5%)	0.68 (0.87)
7.With getting ready for the new baby	240 (43.1%)	17 (3.1%)	0.95 (1.06)

a= Only upper (ceiling) or lower (floor) responses are displayed per item.
M = Mean/SD = Standard Deviation

Supplementary Table S4. Floor and ceiling scores, Means and Standard Deviation for each item in the Interference Scale(n=557).
