# Supplementary material for: Validation and Psychometric Properties of the Spanish Version of the Fear of Childbirth Questionnaire (CFQ-e)
Source: J Clin Med. 2022 Mar 26;11(7):1843. doi: 10.3390/jcm11071843 (PMC8999905; doi:10.3390/jcm11071843)
Supplement: Supplementary file 1 [file jcm-11-01843-s001.zip › TABLE S5. Factor loadings 4 factors 40 items.pdf]

	F1	F2	F3	F4
Item 1	0.091	0.087	0.049	**0.504**
Item2	0.106	-0.039	**0.305**	**0.419**
Item3	0.091	-0.015	-0.087	**0.669**
Item4	0.173	**0.442**	-0.146	0.192
Item5	0.173	**0.426**	-0.083	0.059
Item6	-0.051	0.086	-0.025	**0.880**
Item7	**0.482**	0.051	0.040	-0.184
Item8	0.217	-0.026	0.264	0.295
Item9	0.037	-0.023	-0.080	**0.887**
Item10	-0.079	0.078	-0.056	**0.893**
Item11	0.127	0.099	**0.307**	0.140
Item12	**0.509**	-0.142	0.189	0.135
Item13	**0.696**	-0.180	0.024	0.230
Item14	0.201	0.042	0.099	0.213
Item15	**0.769**	-0.010	-0.023	-0.122
Item16	-0.049	-0.054	-0.045	**0.894**
Item17	0.023	0.198	0.129	**0.460**
Item18	0.026	-0.133	**0.548**	**0.350**
Item19	0.063	**0.309**	**0.347**	0.133
Item20	-0.075	-0.000	-0.026	**0.826**
Item21	**0.515**	0.161	0.030	-0.147
Item22	0.130	**0.527**	-0.115	0.073
Item23	**0.463**	**0.316**	0.042	-0.198
Item24	**0.819**	-0.018	0.049	-0.074
Item25	**0.543**	**0.312**	-0.098	-0.141
Item26	**0.693**	0.011	-0.085	0.216
Item27	**0.742**	0.003	-0.184	0.211
Item28	0.188	**0.369**	0.280	-0.014
Item29	-0.024	-0.097	**0.669**	0.206
Item30	-0.041	-0.050	**0.838**	0.018
Item31	0.282	-0.115	**0.497**	-0.013
Item32	**0.517**	0.167	0.100	-0.106
Item33	-0.025	**0.798**	-0.054	-0.022
Item34	-0.106	-0.002	**1.001**	-0.096
Item35	-0.058	0.040	**0.950**	-0.097
Item36	-0.108	**0.892**	0.037	-0.056
Item37	-0.037	-0.057	**0.783**	-0.088
Item38	-0.108	**0.467**	0.295	0.080
Item39	-0.185	**0.559**	0.271	0.214
Item40	0.009	**0.635**	0.049	0.095

Supplementary Table S5. Factor loadings (after rotation) of the model obtained from 4 factors and 40 items of the first versión of CFQ-E on the first sample (n=279)
