# Supplementary material for: Validation and Psychometric Properties of the Spanish Version of the Fear of Childbirth Questionnaire (CFQ-e)
Source: J Clin Med. 2022 Mar 26;11(7):1843. doi: 10.3390/jcm11071843 (PMC8999905; doi:10.3390/jcm11071843)
Supplement: Supplementary file 1 [file jcm-11-01843-s001.zip › TABLE S6 Factor loadings CFA 4factor 40 items.pdf]

Supplementary Table S6 Model factor loadings (after rotation) obtained from 4 factors-40 items from the first version of the CFQ-S in the second sample (n=278)

	F1<br>Fears relating to sexual aspects and embarrassment	F2<br>Fear of Medical Interventions	F3<br>Fear of pain	F4<br>Fear of harm to self and dying
Item 1-Being harmed by incompetent medical care	0.010	0.146	-0.002	**0.571**
Item2-Tears or rectal damage resulting from childbirth	0.164	0.013	0.238	**0.437**
Item3-Dying during labour	-0.234	0.034	0.113	**0.784**
Item4-Receiving general anaesthesia	-0.085	**0.463**	-0.018	0.233
Item5-Receiving injections	0.001	**0.519**	0.064	0.065
Item6-Harm to self or to my baby during labour and birth	-0.086	-0.036	-0.061	**0.913**
Item7- Being seen naked during labour	**0.577**	0.061	-0.063	-0.097
Item8-A vaginal tear during labour	0.248	0.089	0.197	**0.350**
Item9-My baby will be harmed during labour	0.004	-0.027	-0.090	**0.952**
Item10- My baby will be harmed during a medical intervention during labour (e.g., vacuum, anaesthesia, forceps, etc.)	-0.005	0.011	-0.089	**0.907**
Item11-Not having a C-section despite desiring one	0.196	-0.139	0.265	0.244
Item12-My vagina stretching after a vaginal birth	**0.623**	-0.085	0.250	0.014
Item13-Loss of sexual pleasure because of stretched vagina after birth	**0.854**	-0.164	0.014	0.077
Item14-Losing self-control in front of other people (being rude, screaming) during labour	0.150	0.131	0.066	0.208
Item15-A less attractive body after childbirth	**0.690**	0.017	-0.069	0.026
Item16-My baby will suffocate during labour	-0.099	-0.010	-0.078	**0.943**
Item17-Need forceps or vacuum delivery	0.038	0.178	-0.006	**0.483**
Item18-Not receiving adequate pain relief	0.105	-0.162	0.396	**0.475**
Item19-Having an episiotomy (a cut made to the vagina)	**0.311**	0.257	0.096	0.198
Item20-My baby dying during labour	-0.047	-0.008	-0.021	**0.724**
Item21-Being seen urinating during labour	**0.571**	0.084	0.043	-0.100
Item22-Having an epidural	-0.041	**0.668**	-0.040	-0.005
Item23-Feeling observed by strangers during labour	**0.657**	0.159	-0.050	-0.115
Item24-Having a less attractive vagina after a vaginal birth	**0.897**	-0.014	-0.037	-0.132
Item25-Having scars after a C-section	0.368	**0.401**	-0.004	-0.108
Item26-Loss of sexual pleasure because of pain or discomfort after childbirth	**0.685**	-0.005	-0.011	0.152
Item27-My partner loses sexual pleasure after childbirth because of my stretched vagina after childbirth.	**0.762**	-0.044	-0.105	0.113
Item28-Needing stitches after childbirth	0.308	**0.442**	0.095	-0.027
Item29-Not receiving an epidural during labour despite desiring or needing it	0.089	-0.035	**0.465**	0.229
Item30-Feeling pain during labour	0.073	-0.025	**0.862**	0.053
Item31-Having a vaginal birth	0.216	-0.063	**0.512**	0.007
Item32-Being seen defecating during labour	**0.713**	0.138	-0.039	-0.118
Item33-Not proceeding with a vaginal birth preferring one	0.049	**0.768**	-0.121	-0.003
Item34-Feeling pain while pushing	-0.092	-0.011	**0.970**	-0.135
Item35-Feeling pain during a vaginal birth	-0.127	0.035	**0.980**	-0.093
Item36-Having a C-section	-0.122	**0.925**	-0.022	-0.094
Item37-Feeling pain during contractions	-0.105	0.105	**0.864**	-0.089

Item38-Having a catheter (a tube inserted in the bladder to collect urine)	-0.054	**0.527**	0.229	0.083
Item39-Feeling pain during a C-section	-0.056	**0.540**	0.179	0.157
Item40-Not proceeding with the type of childbirth I chose (e.g., vaginal or C-section)	0.023	**0.633**	0.027	0.093
