# Supplementary material for: Validation and Psychometric Properties of the Spanish Version of the Fear of Childbirth Questionnaire (CFQ-e)
Source: J Clin Med. 2022 Mar 26;11(7):1843. doi: 10.3390/jcm11071843 (PMC8999905; doi:10.3390/jcm11071843)
Supplement: Supplementary file 1 [file jcm-11-01843-s001.zip › TABLE S7 Congruence indices 4-factor-40 item EFA.pdf]

Supplementary Material S7. Table of data congruence indices with the semi-specified four-factor-40 item model based on the results of the first sample's EFA.

Variable	Congruence Index	95% Confidence intervals	
ITEM1	0.969	(0.905	0.998)
ITEM2	0.834	(0.708	0.938)
ITEM3	0.948	(0.887	0.989)
ITEM4	0.881	(0.618	0.986)
ITEM5	0.985	(0.940	0.999)
ITEM6	0.993	(0.987	0.998)
ITEM7	0.975	(0.914	0.998)
ITEM8	0.729	(0.565	0.871)
ITEM9	0.995	(0.988	0.999)
ITEM10	0.995	(0.985	1.000)
**ITEM11**	**0.613**	**(0.203**	**0.895)**
ITEM12	0.921	(0.783	0.988)
ITEM13	0.978	(0.911	0.997)
**ITEM14**	**0.705**	**(0.410**	**0.963)**
ITEM15	0.994	(0.984	1.000)
ITEM16	0.991	(0.982	0.999)
ITEM17	0.936	(0.835	0.990)
ITEM18	0.951	(0.849	0.994)
**ITEM19**	**0.208**	**(-0.165**	**0.531)**
ITEM20	0.997	(0.995	1.000)
ITEM21	0.972	(0.901	1.000)
ITEM22	0.996	(0.994	1.000)
ITEM23	0.839	(0.617	0.961)
ITEM24	0.988	(0.978	0.997)
ITEM25	0.980	(0.957	1.000)
ITEM26	0.976	(0.952	0.995)
ITEM27	0.978	(0.942	0.999)
ITEM28	0.693	(0.437	0.890)
ITEM29	0.883	(0.718	0.975)
ITEM30	0.994	(0.982	1.000)
ITEM31	0.916	(0.731	0.995)
ITEM32	0.968	(0.904	0.998)
ITEM33	0.986	(0.915	0.999)
ITEM34	0.986	(0.967	0.998)
ITEM35	0.987	(0.962	0.997)
ITEM36	0.986	(0.961	0.999)
ITEM37	0.980	(0.950	0.998)
ITEM38	0.917	(0.817	0.988)
ITEM39	0.911	(0.768	0.995)
ITEM40	0.988	(0.970	0.998)
