# Supplementary material for: Validation and Psychometric Properties of the Spanish Version of the Fear of Childbirth Questionnaire (CFQ-e)
Source: J Clin Med. 2022 Mar 26;11(7):1843. doi: 10.3390/jcm11071843 (PMC8999905; doi:10.3390/jcm11071843)
Supplement: Supplementary file 1 [file jcm-11-01843-s001.zip › TABLE S8. Root Mean Square Discrepancy (RMSD).pdf]

Supplementary Table S8. Root Mean Square Discrepancy (RMSD) between rotated loading matrix and target matrix

	RMSD	95% Confidence intervals	
ITEM1	0.084	(0.014	0.141)
ITEM2	0.174	(0.109	0.230)
ITEM3	0.140	(0.060	0.212)
ITEM4	0.137	(0.046	0.199)
ITEM5	0.037	(0.010	0.042)
ITEM6	0.054	(0.022	0.077)
ITEM7	0.086	(0.009	0.166)
ITEM8	0.192	(0.139	0.226)
ITEM9	0.053	(0.014	0.097)
ITEM10	0.054	(0.010	0.097)
ITEM12	0.139	(0.057	0.223)
ITEM13	0.108	(0.036	0.195)
ITEM15	0.042	(0.003	0.070)
ITEM16	0.062	(0.026	0.090)
ITEM17	0.111	(0.041	0.165)
ITEM18	0.132	(0.032	0.241)
ITEM20	0.022	(0.003	0.030)
ITEM21	0.072	(0.013	0.125)
ITEM22	0.050	(0.016	0.079)
ITEM23	0.125	(0.021	0.247)
ITEM24	0.067	(0.027	0.095)
ITEM25	0.067	(0.004	0.123)
ITEM26	0.101	(0.048	0.139)
ITEM27	0.100	(0.032	0.153)
ITEM28	0.185	(0.102	0.243)

ITEM29	0.151	(0.090	0.212)
ITEM30	0.054	(0.009	0.093)
ITEM31	0.130	(0.028	0.224)
ITEM32	0.101	(0.027	0.186)
ITEM33	0.088	(0.022	0.201)
ITEM34	0.078	(0.033	0.122)
ITEM35	0.077	(0.025	0.139)
ITEM36	0.083	(0.008	0.126)
ITEM37	0.087	(0.017	0.149)
ITEM38	0.131	(0.065	0.215)
ITEM39	0.131	(0.064	0.204)
ITEM40	0.059	(0.008	0.088)
