# Supplementary material for: Validation and Psychometric Properties of the Spanish Version of the Fear of Childbirth Questionnaire (CFQ-e)
Source: J Clin Med. 2022 Mar 26;11(7):1843. doi: 10.3390/jcm11071843 (PMC8999905; doi:10.3390/jcm11071843)
Supplement: Supplementary file 1 [file jcm-11-01843-s001.zip › TABLE S9 Final version CFQ-e 4 subscales-37 items SPANISH ENGLISH VERSION.pdf]

Supplementary Table S9. Final version of the CFQ-e (English and Spanish version)

**Subscales and items for the final CFQ-e Model (4 subscales-37 items)**
Fear relating to sexual aspects and embarrassment Subscale
12- My vagina stretching after a vaginal birth
13- Loss of sexual pleasure because of stretched vagina after childbirth
15- A less attractive body after childbirth
24-A less attractive vagina after vaginal childbirth
26- Loss of sexual pleasure because of pain or discomfort after childbirth
27- My partner loses sexual pleasure after childbirth because of my stretched vagina after childbirth.
7 Being seen naked during labour
21- Being seen urinating during labour
23-Feeling observed by strangers during labour
32-Being seen defecating during labour
Fear of Medical Interventions Subscale
4-Receiving general anaesthesia
5-Receiving injections
22-Having an epidural
28-Needing stitches after childbirth
33- Not proceeding with a vaginal birth despite preferring one
36-Having a C-section
38-Having a catheter (a tube that is inserted in the bladder to collect urine
39-Feeling pain during a C-section
40- Not proceeding with the type of childbirth I chose (e.g., vaginal or C-section)
25- Having scars after a C-section
Fear of pain Subscale
29- Not receiving an epidural during labour despite desiring or needing one
30-Feeling pain during labour
31-Having a vaginal childbirth
34-Feeling pain while pushing
35-Feeling pain during a vaginal birth
37-Feeling pain during contractions
18-Not receiving adequate pain relief
Fear of harm and dying Subscale
1- Being harmed by incompetent medical care
2-Tears or rectal damage resulting from childbirth
3-Dying during labour
6-They cause injury or harm to my baby due to the delivery
8- A vaginal tear during labour
9 My baby will be harmed during labour
10 They harm the baby in the course of a medical intervention during labour (e.g., vacuum, anaesthesia, forceps, etc.)
16-My baby suffocating during labour
17-Needing a forceps or vacuum
20-My baby dying during labour
Items removed from the final version of the CFQ-e with respect to the original CFQ version
11-Not having a C-section despite desiring one
14-Losing self-control in front of other people (being rude, screaming) during labour
19-Have an episiotomy performed (have a cut made in your vagina)

Subescalas e items para el Modelo final del CFQ-e
Subescala Miedo respecto aspectos sexuales y vergüenza
12-Que tu vagina se estire por tener un parto vaginal
13-Disfrutar menos de las relaciones sexuales por el estiramiento de la vagina a causa del parto vaginal
15-Que tu cuerpo sea menos atractivo tras el parto
24-Que tu vagina se vea menos atractiva tras un parto vaginal
26-Disfrutar menos de las relaciones sexuales por sentir dolor o molestias tras el parto
27-Que tu pareja disfrute menos de las relaciones sexuales después del parto por el estiramiento de tu vagina tras el parto
7 Que otras personas te vean desnuda durante el parto
21-Que otras personas te vean orinar durante el parto
23-Sentirte observada por desconocidos durante el parto
32-Que otras personas te vean defecar durante el parto
Subescala Miedo a Procedimientos médicos
4-Recibir anestesia general
5-Que te administren inyecciones
22-Que te administren la epidural
28-Necesitar puntos de sutura tras el parto
33-No poder tener un parto vaginal a pesar de ser lo que prefieres
36-Tener una cesárea
38-Que te sonden (un tubo que se inserta en la uretra para recoger la orina
39-Sentir dolor durante una cesárea
40-No poder tener el tipo de parto que te gustaría (por ejemplo vaginal o cesárea)
25-Que te queden cicatrices tras la cesárea
Subescala Miedo al dolor
29-Que no te pongan la epidural durante el parto en el caso de quererla o necesitarla
30-Sentir dolor durante el parto
31-Tener un parto vaginal
34-Sentir dolor mientras empujas al bebe
35-Sentir dolor durante un parto vía vaginal
37-Sentir dolor durante las contracciones
18-No recibir la medicación para el dolor que necesitas
Subescala Miedo al daño corporal y a la muerte
1-Que te hagan daño por una asistencia médica incompetente
2-Sufrir un desgarro o daño rectal como consecuencia del parto
3-Morir durante el parto
6-Que dañen o perjudiquen al bebe como consecuencia del parto
8-Sufrir un desgarro vaginal durante el parto
9 Que el bebe sufra algún daño durante el parto
10 Que dañen al bebe en una intervención médica durante el parto (ej, ventosa, anestesia, fórceps ...)
16-Que el bebe se asfixie durante el parto
17-Necesitar un fórceps o ventosa
20-Que el bebe muera durante el parto
Items eliminados respecto a la version original del CFQ
11-Que no te hagan una cesárea cuando es lo que tú quieres
14-Perder el control de tus emociones frente a otras personas (ser maleducada, gritar) durante el parto
19-Que te realicen una episiotomía (que te realicen un corte en tu vagina)
